# *Leishmania infantum*-derived lipophosphoglycan as an antigen in the accurate serodiagnosis of canine leishmaniasis

**DOI:** 10.1371/journal.pntd.0007720

**Published:** 2019-09-12

**Authors:** Ricardo Dias Portela, Rodrigo Pedro Soares, Gabriela Porfírio Passos, Daniela Farias Larangeira, Thiago Doria Barral, Julia Ramos Sampaio, Marcos F. Bernardo, Edneia Venâncio Alves-Sobrinho, Maria Terezinha Bahia, Flaviane Alves Pinho, Stella Maria Barrouin-Melo

**Affiliations:** 1 Laboratory of Immunology and Molecular Biology, Institute of Health Sciences, Federal University of Bahia (UFBA); Salvador, Bahia; Brazil; 2 Laboratory of Cellular and Molecular Parasitology, René Rachou Institute, Oswaldo Cruz Foundation; Belo Horizonte, Minas Gerais; Brazil; 3 Laboratory of Veterinary Infectious Diseases, Teaching Hospital of Veterinary Medicine, UFBA; Salvador, Bahia; Brazil; 4 Department of Anatomy, Pathology, and Veterinary Clinics of the School of Veterinary Medicine and Zootechny, UFBA, Salvador, BA, Brazil, CEP; 5 Institute of Exact and Biological Sciences, Federal University of Ouro Preto, Campus Morro do Cruzeiro; Ouro Preto, Minas Gerais; Brazil; Institut Pasteur de Tunis, TUNISIA

## Abstract

Lipophosphoglycan (LPG) is the major surface glycoconjugate of *Leishmania* protozoan and has an important biological role in host-parasite interactions both in the midgut epithelium of the sand fly vector and in the vertebrate macrophages. Canine leishmaniasis (CanL) is a chronic infectious disease predominantly caused by *Leishmania infantum*. An early and accurate immunodiagnosis of the disease is crucial for veterinary clinical practice and for disease control. In this work, we evaluated *L*. *infantum* LPG as an antigen in an indirect enzyme-linked immunosorbent assay (ELISA) for CanL immunodiagnosis (LPG-ELISA) by testing serum samples from 97 naturally infected dogs with diverse clinical presentations ranging from subclinical infection to severe disease, as evaluated by veterinarian infectologists. Serum samples from healthy dogs from non-endemic areas (n = 68) and from dogs with other infectious diseases (n = 64) were used as controls for assay validation. The performance of the LPG-ELISA was compared with that of an ELISA using the soluble fraction of *L*. *infantum* total lysate antigen (TLA). LPG-ELISA presented a superior performance in comparison to TLA-ELISA, with 91.5% sensitivity, 98.5% specificity and 99.7% accuracy. A distinguishing feature of the LPG-ELISA compared to the TLA-ELISA was its higher ability to identify subclinical infection in clinically healthy dogs, in addition to the absence of cross-reactivity with other canine infectious diseases. Finally, LPG-ELISA was compared to TR DPP visceral canine leishmaniasis test, the immunochromatographic test recommended by the Brazilian Ministry of Agriculture. LPG-ELISA exhibited higher values of specificity (98.5% versus 93.1%) and sensitivity (91.5% versus 90.6%) compared to TR DPP. In conclusion, *L*. *infantum*-derived LPG was recognized by antibodies elicited during CanL in different infection stages and was shown to be a suitable antigen for specific clinical settings of veterinary diagnosis and for public health usage.

## Author summary

Canine leishmaniasis (CanL) caused by *Leishmania infantum* is a zoonotic disease with high importance for the public health of several countries. *L*. *infantum*-infected dogs can be a domestic reservoir of the protozoan parasite for sand flies, which transmit it from dogs to humans during their blood meals. It is important to diagnose and treat infected dogs as early as possible, so the dissemination of CanL and the transmission to humans can be controlled. The currently commercially available assays present problems, such as cross-reaction with other canine diseases and lack of sensitivity in the detection of dogs that do not present clinical signs of disease. In this work, we tested lipophosphoglycan (LPG), a molecule abundantly found in the protozoan´s surface, as an antigen in an immunodiagnostic platform. We were able to see that the assay using LPG is highly sensitive and specific, showed no cross-reaction with other canine infectious diseases and successfully identified infected dogs with no signs of disease.

## Introduction

Leishmaniasis comprehend a complex of chronic zoonotic diseases caused by intracellular protozoa from the *Leishmania* genus. The etiologic agent of visceral leishmaniasis in India and East Africa is *Leishmania donovani*, while the disease is predominantly caused by *Leishmania infantum* in the Middle East, central Asia, Mediterranean countries and the Americas [1]. In Latin America and Mediterranean countries, dogs (*Canis familiaris*) with canine leishmaniasis (CanL) are the main sources of *L*. *infantum* infection for the invertebrate sand fly vector [2].

*Leishmania* has a great ability to evade the host immune system. To survive in the host’s mononuclear phagocytic cells, this parasite developed biochemical and morphological adaptations, and glycoconjugates are the main molecules involved in these processes [3]. The most studied *Leishmania* glycoconjugate is the lipophosphoglycan (LPG), a dense glycocalix covering the promastigote’s surface and flagellum [4]. Its carbohydrate motif shares similarities with the proteophosphoglycans (PPGs) found in the intracellular amastigote stage, which causes disease in vertebrate hosts [5, 6]. LPGs mediate several mechanisms that are essential to parasite virulence, both in the vertebrate and invertebrate host, such as immunomodulation and attachment to the sand fly midgut, respectively [7, 8].

Several immunodiagnostic tests for CanL have been developed to detect specific antibodies against *Leishmania*. In Brazil, the Health Ministry recommends an immunochromatographic assay based on a recombinant rK28 antigen of *L*. *infantum* (TR DPP -CVL) combined with an indirect ELISA based on *L*. *major* crude total antigen (EIE) as criteria for the culling of seropositive dogs in surveillance and control programs for visceral leishmaniasis [9]. Although those tests present good sensitivity in the detection of diseased dogs (“symptomatic dogs”, as called by some authors), they cannot distinguish susceptible and resistant dogs and are quite insensitive for the detection of subclinical infection in clinically healthy dogs (also called by some authors as “asymptomatic dogs”). Thus, assays with better predictive values are still needed [10, 11]. Moreover, many serologic assays can present false-positive results due to cross reactions with other species of *Leishmania* such as *L*. *braziliensis* [12], and with other infectious agents that are common in dogs, including *Trypanosoma cruzi* [13], *Ehrlichia canis*, *Babesia canis* [14, 15].

The search for an ideal *Leishmania* antigen for CanL immunodiagnosis has focused on proteins and peptides, most of which are found in proteomic studies [16, 17]. However, disadvantages of the use of such molecules include the time-demanding and high-cost processes and the low stability of the synthetized compounds. The use of glycoconjugates as antigens has not been fully explored [18, 19], but the ability of LPG to stimulate high antibody titers in human hosts has also been demonstrated [20]. Moreover, scientific evidence of the high stability of these molecules and a high yield of the purification procedures [21] suggests that they can be advantageous for immunotests. Thus, since *Leishmania*-derived glycoconjugates have immunomodulatory and immunogenic properties and has been described as having a similar structure in *L*. *infantum* isolates from different continents [22], phosphoglycans (PGs) could be promising candidates for the immunodiagnosis of the infection. In this context, the present study was designed to evaluate the application of LPG as an antigen for CanL immunodiagnosis.

## Methods

### Ethics statement

The present study was approved by the Committee on Ethical Use of Experimental Animals of the School of Veterinary Medicine of the Federal University of Bahia (n. 023/2013). All procedures involving animals were conducted according to the guidelines of the Brazilian Council of Animal Experimentation (CONCEA) and strictly followed the Brazilian law for “Procedures for the Scientific Use of Animals” (11.794/2008).

### Animals and biological samples

Serum samples from dogs with confirmed diagnosis of *L*. *infantum* infection as well as samples from non-endemic areas for leishmaniasis were used to standardize the ELISAs. Ninety-seven dogs were diagnosed as infected by *L*. *infantum* DNA detection by PCR in blood, skin biopsy or aspirates from lymph node, bone marrow or spleen; parasitological assays were carried out using tissue aspirates. *L*. *infantum*-infected dogs were used as positive controls in this study. Among the selected positive dogs, 10 were clinically healthy by means of physical and clinical pathology exams, and 87 presented a variable range of disease, from mild to severe CanL (**Table 1**). Sixty-eight canine serum samples from non-endemic areas were included as negative controls, and those dogs should not have history of traveling to endemic areas; dogs from non-endemic areas were evaluated by the same molecular and parasitological tests as those for the positive control dogs (**Table 1**). In addition, sera from 30 dogs from an endemic area, presenting negative results in molecular and parasitological assays, were included in the study. The exclusion criteria were lack of confirmation of *L*. *infantum* infection by molecular or parasitological methods or diagnosis of any coinfections. All dogs also underwent blood and urine sampling for hemogram, serum biochemistry tests and urinalysis.

**Table 1:** Characterization of the dogs from which the serum samples used in this experiment were obtained. The *L*. *infantum* negative and positive controls were used for the ELISA standardization and establishment of the validation parameters. The serum samples taken from dogs infected with other pathogens than *L*. *infantum* were used for the cross-reactivity assay.

Animal characteristics	N	Diagnostic assay
Dogs infected by *Leishmania infantum* (positive controls)	97	Culture and PCR
Dogs from *L*. *infantum* non-endemic areas (negative controls)	68	Culture and PCR
Dogs infected by *Leishmania braziliensis* (subclinical infection)	07	Culture and PCR
Dogs from an endemic area and negative in molecular and parasitological assays	30	Culture and PCR
Dogs infected by *L*. *braziliensis* (diseased)	06	Culture and PCR
Dogs experimentally infected by *Trypanosoma cruzi* (acute phase)	10	—
Dogs experimentally infected by *T*. *cruzi* (chronic phase)	10	—
Dogs infected by *Hepatozoon* sp.	06	Cytology and PCR
Dogs infected by *Ehrlichia* sp.	11	Cytology and PCR
Dogs infected by *Babesia* sp.	08	Cytology and PCR
Dogs infected by *Anaplasma* sp.	07	Cytology and PCR
Dogs infected by 2 or 3 of the above pathogens at the same time	05	Cytology and PCR

Samples for parasitological and molecular diagnostic exams of the studied dogs were obtained under sedation with acepromazine (0.1 mg/kg; intravenous). Fine needle aspirates were obtained as previously described from the spleen [23], bone marrow and/or lymph nodes and immediately processed or kept at -20°C.

Sera from dogs with infections other than by *L*. *infantum* were kindly provided by colleagues from other research institutions and used for specificity assay determinations. Thirteen of those samples were from dogs infected with *L*. *braziliensis*. Twenty samples were from *T*. *cruzi* experimentally infected dogs and collected in the acute and chronic phases of disease. Thirty-one samples from dogs with other pathogens, whose infections were confirmed by molecular or parasitological tests, were obtained from non-endemic *L*. *infantum* areas (**Table 1**).

### Clinical staging of *Leishmania*-infected dogs

All of the 97 dogs naturally infected by *L*. *infantum* underwent a clinical evaluation by physical examination and clinical pathology for CanL severity staging as proposed by Solano-Gallego et al. [24, 25]. Accordingly, infected dogs were characterized into Stage 1 –clinically healthy parasitized dogs (n = 10); Stage 2 –dogs with mild clinical disease (n = 23); Stage 3 –dogs with severe disease (n = 56); and Stage 4 –dogs presenting very severe clinical disease (n = 68). Hemograms were carried out using an automatized counter (Sysmex pocH-100iVDiff, Roche), and serum biochemistry analysis was performed using automatized equipment and commercial kits (Wiener) for the quantification of urea, creatinine, total protein, albumin, and urinary protein and creatinine for calculating the urinary protein/creatinine ratio.

### Parasitological diagnosis of *Leishmania* infection

Cytological examinations were performed in smears of freshly obtained fine needle aspirates of bone marrow and/or lymph nodes. Smears were stained with a modified Romanowsky staining rapid test kit (Panótico Rápido, Laborclin, Brazil) and analyzed under optical microscopy. A positive cytological result was given by the finding of amastigote forms within cells.

For isolation of *Leishmania* sp. in culture medium, freshly obtained spleen aspirates were inoculated into biphasic Novy-MacNeal-Nicolle (NNN) medium with Schneider’s liquid phase (Sigma-Aldrich Inc., USA) supplemented with 20% bovine fetal serum (Gibco, USA) and 50 μg/mL gentamicin, as standardized previously [23, 26]. The cultures were kept in a BOD chamber at 23°C and examined weekly under optical microscopy for 30 days. Positive culture results were identified by the visualization of moving promastigote forms.

### DNA extraction and PCR for *L*. *infantum* diagnosis

Molecular diagnosis was performed to confirm that the natural infection of the studied dogs was in fact due to *L*. *infantum* by detecting the parasite’s DNA in their biological samples. Thus, genomic DNA was extracted from *Leishmania* cultures isolated from the dogs as well as from blood samples or spleen, bone marrow or lymph node fine needle aspirates, using a commercial kit (Wizard Genomic DNA Purification Kit, Invitrogen Life, Brazil), following the manufacturer’s recommendations. Purified DNA was then tested by the PCR methodology proposed by Lachaud et al. [27] using the primers RV1 (forward; 5’-CTTTTCTGGTCCCGCGGGTAGG-3’) and RV2 (reverse; 5’-CCACCTGGCCTATTTTACACCA-3’), which amplify *L*. *infantum* kinetoplast DNA.

### *L*. *infantum*-derived LPG isolation and purification

Promastigotes of *L*. *infantum* (MCAN/BR/89/Ba-262) were washed in PBS and centrifuged at 2,100 x *g*. The pellet was mixed with 2.5 mL of a CHCl_3_/MeOH (3:2) solution and 0.5 mL of 4 mM MgCl_2_. The mixture underwent sonication and centrifugation, and this procedure was repeated twice. The resulting pellet was treated with 3.0 mL of CHCl_3_/MeOH/H_2_0 (10:10:3) and 0.5 mL of CHCl_3_/MeOH (1:1). For the LPG extraction, 2.5 mL of ESOAK (water/ethanol/ethyl ether/pyridine/NH_4_OH 15:15:5:1:0.017) was added to the resulting pellet, followed by sonication and centrifugation. The supernatant containing LPG was then evaporated with nitrogen and resuspended in 1 mL of CH_3_COOH 0.1 N/NaCl 0.1 N. This final solution was applied to a phenyl-sepharosis column (BIO-RAD #731–1550). After inoculating the solution containing LPG, the column was washed with six volumes of CH_3_COOH 0.1 N/NaCl 0.1. After one additional wash with 1 mL of CH_3_COOH 0.1 N and one wash with 1 mL of ddH_2_0, 4 mL of ESOAK was used for LPG elution. The eluate was then evaporated with nitrogen and resuspended in 1 mL of ultrapure water. To confirm the presence of LPG in the final eluate, the CA7AE monoclonal antibody was used for western blotting as reported previously [22].

### *Leishmania infantum* total lysate antigen (TLA) production

*L*. *infantum* cultures (MCAN/BR/89/Ba-262) were expanded in biphasic NNN-Schneider medium (Sigma-Aldrich Inc., USA) supplemented with 20% fetal bovine serum and 50 μg/mL gentamicin and kept in a BOD chamber at 23°C until reaching stationary phase. The culture was then centrifuged at 3,000 x g for 10 minutes and the parasite pellets washed three times with sterile PBS before storage at -80°C. For antigen purification, the parasite pellets were submitted to three cycles of ultrasound at 4 Hz for one minute and then centrifuged at 14,000 x *g* for 10 minutes at 4°C. The supernatant was then collected, and the protein concentration determined by the bicinchoninic acid method (Thermo Fisher, Waltham, MS).

### Western blotting analysis

Western blotting was carried out to assess the recognition of LPG by antibodies from *L*. *infantum-*infected dogs. A purified LPG solution was submitted to an electrophoretic run on a 12.5% polyacrylamide gel (SDS-PAGE) and then transferred to nitrocellulose membranes. After a blocking step with 10% casein in PBS pH 7.4 for 16 hours at -4°C, the membranes were washed three times with PBS with 0.05% Tween 20 (PBST) and incubated with pool of control sera (diluted 1: 1,000 in PBST with 5% casein) for two hours under agitation at 23° C. After three more washes with PBST, the membranes were incubated with a horseradish peroxidase-conjugated anti-dog IgG antibody (Bethyl, USA) under agitation for one hour. After three more washes with PBST, the membranes were incubated with an enzyme substrate and chromogen solution (4-chloro-1-naphtol and hydrogen peroxide) for ten minutes, and the reaction was stopped with ultrapure water.

### Development and standardization of LPG-ELISA and TLA-ELISA

The ELISAs were standardized by a checkerboard titration method. Different antigen (LPG and TLA) concentrations and different dilutions of the control pooled sera and of the anti-canine IgG antibody conjugated to horseradish peroxidase were tested. The optimal assay conditions were determined by the higher ratio value between the positive and negative pool’s OD readings. The positive and negative serum pools consisted of an equal quantity of ten negative or ten positive control serum samples, as described in **Table 1**.

The ELISA was developed on high-binding flat bottom polystyrene microplates (Parker Elmer, Waltham USA), which were sensitized with LPG or TLA diluted in 100 μL carbonate/bicarbonate buffer pH 9.6 per well at 4°C for 14 hours. The plates were then washed two times with PBS 0.05% Tween 20 (PBST) blocked with casein 10% in PBS and incubated at 37°C for three hours. After four more washes with PBST, 100 μL of serum samples diluted in PBST with 5% of casein were added in duplicate to the wells and incubated at 37°C for one hour. The plates were washed six times with PBST, and the anti-canine IgG horseradish peroxidase antibody (Bethyl, USA) diluted in PBST with 0.5% casein was added and incubated for one hour at 37°C. After six washes, the reaction was developed with a solution containing ortophenylenediamine (OPD) and peroxide hydrogen, diluted in citrate buffer pH 4.3 and interrupted with H_2_SO_4_ 4N. The OD readings were obtained using an ELISA plate reader (BIO-RAD, USA).

### Comparison of LPG-ELISA with an immunochromatographic rapid assay

The positive and negative controls that were used for the LPG-ELISA standardization and the serum samples from dogs naturally infected with *L*. *braziliensis* or experimentally infected with *T*. *cruzi* were tested by the TR DPP-CVL test (Bio-Manguinhos, Rio de Janeiro, Brazil). This test consists in a validated dual-path immunochromatographic platform using the rK28 recombinant protein as antigen, and is currently recommended as the official screening test by the Brazilian Ministry of Agriculture [28]. All the samples were tested as recommended by the manufacturer.

### Statistical analysis

The ELISA cut-off was defined as the mean OD of negative serum samples plus three standard deviations [29]. The calculations of specificity, sensitivity, negative and positive predictive values (NPV and PPV) were based on the number of positive (for *L*. *infantum* or other infections) and negative control serum samples that presented positive or negative results in each ELISA [30]. A receiver operating characteristics (ROC) curve was obtained for each ELISA using SPSS v. 12.0. software (IBM, USA), and the accuracy was defined as the area under the curve (AUC) of each ROC curve. The agreement between ELISA results and parasitology/molecular positivity for *L*. *infantum* infection was calculated using the Kappa (K) index with the following classification: 0 –no concordance; 0 to 0.19 –very low correlation; 0.20 to 0.40 –weak correlation; 0.40 to 0.59 –moderate concordance; 0.60 to 0.79 –substantial concordance; 0.80–1.00 –high concordance [31]. The calculation of repeatability and reproducibility was based on the OD readings obtained in assays carried out by three different technicians in three different days (reproducibility) or in twenty repetitions performed by the same technician at the same time (repeatability). The results for these two parameters were expressed as the percentage (%) of coherent results according to the serum pool infectivity status.

## Results

### LPG is specifically recognized by serum samples taken from *L*. *infantum*-infected dogs

**Fig 1:**
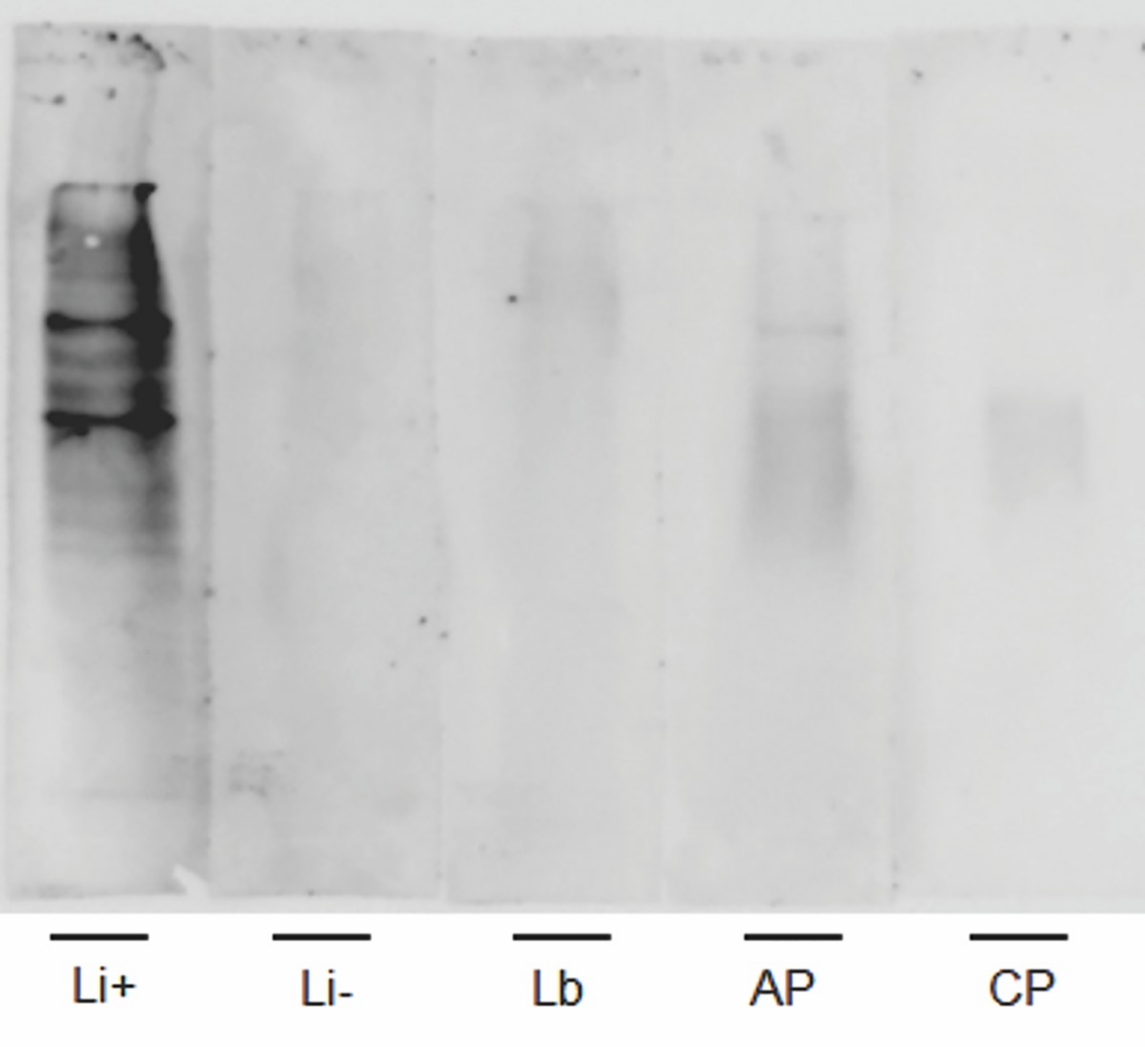
Recognition of LPG by a pool of serum samples taken from dogs with confirmed *Leishmania infantum* infection. The LPG solution was separated using a SDS-PAGE system, transferred to nitrocellulose paper and incubated with (Li+) a pool of serum samples from dogs with a confirmed *L*. *infantum* infection, (Li-) a pool of serum samples from non-endemic area dogs, a pool of serum samples from dogs with a confirmed *L*. *braziliensis* infection, and pools of serum samples that were experimentally infected with *T*. *cruzi* and in the (AP) acute phase or (CP) chronic phase of the infection.

Immunoblot analysis with pooled sera from *L*. *infantum* naturally infected dogs revealed immunoreactivity against the LPG antigen. The pool of positive sera recognized the expected LPG molecule, displaying the expected smear characteristic of glycoconjugates (**Fig 1**). There was no reaction of the transferred LPG to the nitrocellulose membrane with the negative canine pooled sera, sera from dogs in the acute (AP) and chronic (CP) phases of an experimental infection with *T*. *cruzi*, and naturally infected with *L*. *braziliensis* (Lb).

### Standardization of the LPG-ELISA

For the evaluation of LPG as a serodiagnostic antigen candidate for *L*. *infantum* infection, positive canine sera from endemic areas for CanL and negative canine sera from non-endemic areas for the disease were individually tested by ELISA. Each sample was tested against purified LPG and crude extract from *L*. *infantum*. First, all ELISA procedures were optimized with regard to antigen concentrations, control sera and conjugated antibody dilutions by checkerboard titration. LPG-ELISA tests revealed that 0.5 μg/mL of antigen and serum and conjugated antibody dilutions of 1: 400 and 1: 10,000, respectively, produced the best resolution between optical density (OD) readings of the positive and negative sera pools. Similarly, optimal conditions for the TLA-ELISA reaction were achieved using 8.0 μg/mL of antigen and serum and conjugated antibody dilutions of 1: 800 and 1: 10,000, respectively. These conditions achieved a positive: negative OD reading ratio for the serum sample pools of 17.99 for the LPG-ELISA and 13.5 for the TLA-ELISA.

**Fig 2:**
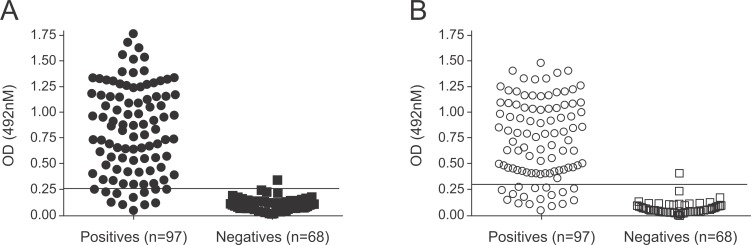
**Distribution of the individual optical density results of the negative and positive controls that were tested by the (A) LPG-ELISA or (B) TLA-ELISA.** The lines within the graphs represent the cut-off of each assay.

Ninety-seven positive serum samples and 68 negative control serum samples were used to evaluate the performance of the LPG antigen in the serological assays (**Fig 2**). The cut-off point, calculated by the mean OD of the negative samples plus three standard deviations, was determined for the LPG- and TLA-ELISA as 0.251 and 0.288, respectively. Both ELISAs identified only one of the negative control samples as positive. Regarding false-negative results, the LPG-ELISA identified nine samples from infected dogs as negative **(S1 Table)**, with OD readings below the established cut-off point, while the TLA-ELISA presented 17 false-negative results.

### LPG-ELISA presents high specificity and sensitivity in the detection of *L*. *infantum*-infected dogs

**Fig 3:**
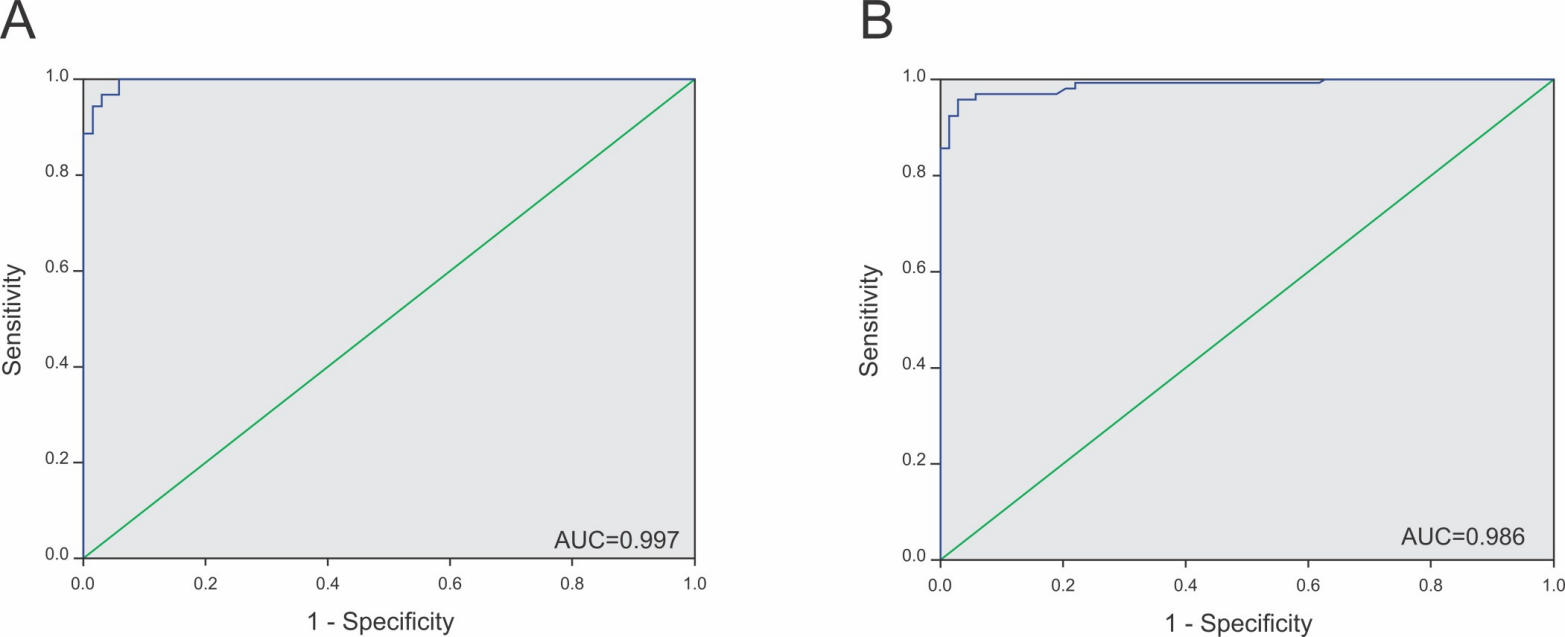
**ROC curves constructed with the results of the positive and negative serum sample controls tested by the (A) LPG-ELISA or (B) TLA-ELISA.** Overall, 97 positive and 68 negative controls were tested in each assay. The curves were made using SPSS v.12.0 software.

As shown in **Table 2**, the diagnostic specificity of the LPG-ELISA was determined as 98.5%, equal to that achieved by the TLA-ELISA (98.5%). However, the LPG-ELISA sensitivity value (91.5%) was superior to that obtained by the TLA-ELISA (85.0%). Both assays were very stable, as they presented high repeatability (97.5% for the LPG-ELISA and 96.1% for the TLA-ELISA) and reproducibility (99.7% for the LPG-ELISA and 95.1% for the TLA-ELISA). The predictive values, which express the possibility of a sample with a specific result being identified with that exact profile, were similar in both assays when considering the positive predictive value (PPV– 98.9%), but the negative predictive value (NPV) was higher for the LPG-ELISA (89.3%) than for the TLA-ELISA (84.8%). The accuracy of each test was calculated using the area under the ROC curve (**Fig 3**), and this parameter was also higher for the LPG-ELISA (99.7%) than for the TLA-ELISA (98.6%). When serum samples taken from dogs from an endemic area and presenting negative results in parasitological and molecular assays were tested by LPG-ELISA, as an example of local cut-off development and application in an endemic area, a 0.310 cut-off could be established. This result made the LPG-ELISA sensitivity drop to 84.6%, considering 15 false-negative results in a total of 97 positive samples tested **(S1 Fig).**

**Table 2:** Results from the canine visceral leishmaniasis positive and negative controls tested by the LPG and TLA ELISAs and consequent validation parameters. These parameters were calculated using the results found at each assay made with the optimized conditions. The accuracies were calculated as the areas under the ROC curves (AUC).

Parameters	ELISA-LPG	ELISA-ATL
Number of samples tested	165	165
Number of positive controls	97	97
Number of negative controls	68	68
True positives	88	80
True negatives	67	67
False-negative results	09	17
False-positive results	01	01
Cut-off	0.251	0.288
Sensitivity (%)	91.5	85.0
Specificity (%)	98.5	98.5
Accuracy (%)	99.7	98.6
Positive predictive value (PPV) (%)	98.9	98.9
Negative predictive value (NPV) (%)	89.3	84.8
Kappa value	0.9	
Repeatability (%)	97.5	96.1
Reproducibility (%)	98.5	95.1

### LPG-ELISA presents no cross-reaction with immunoglobulins from dogs infected with other trypanosomatids or other canine infectious agents

**Fig 4:**
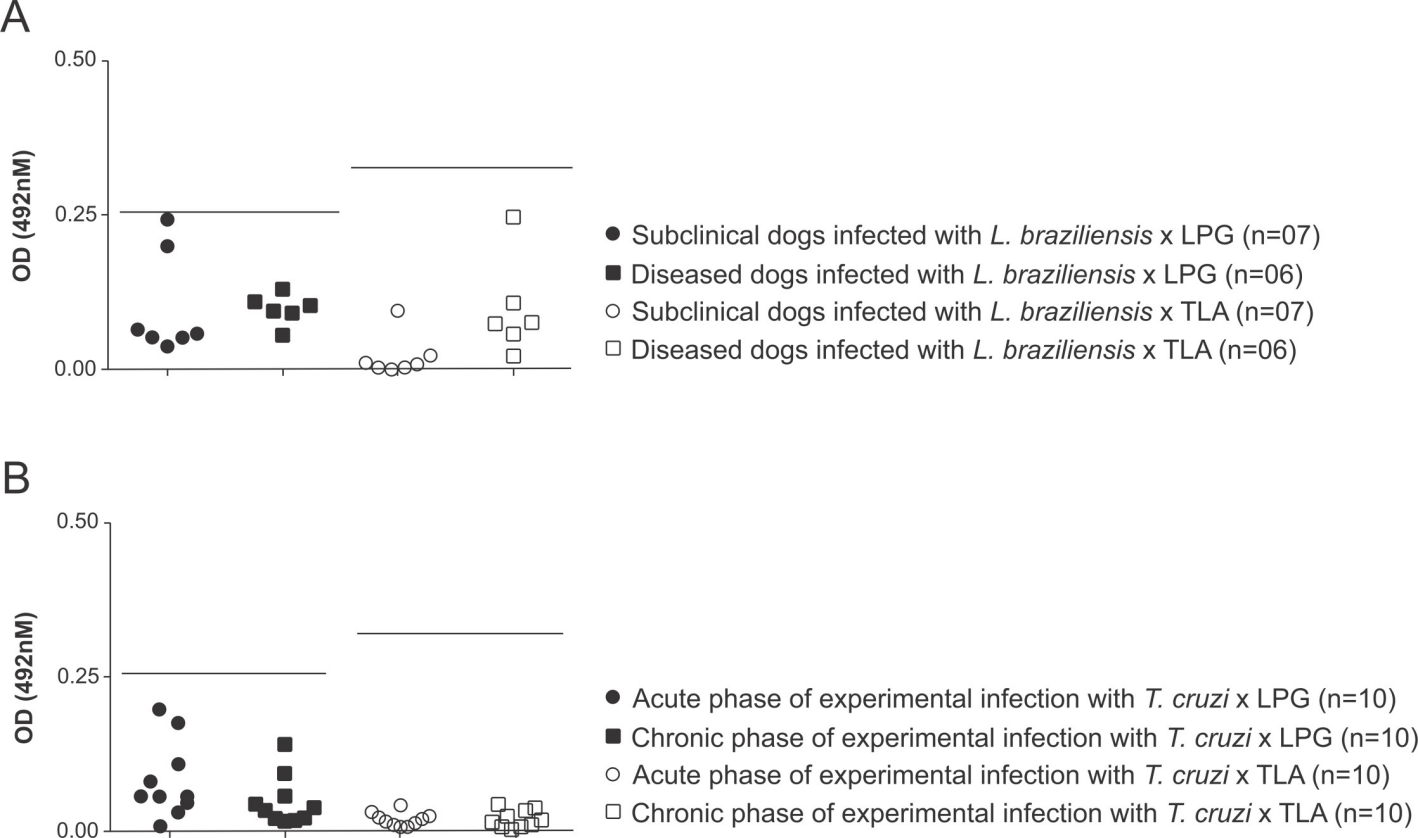
Evaluation of the occurrence of cross-reactivity using the LPG and TLA-ELISAs with samples from dogs infected with other trypanosomatids. Serum samples from **(A)** dogs infected with *L*. *braziliensis* (with or without clinical disease) and **(B)** from dogs experimentally inoculated with *T*. *cruzi* (chronic and acute phases of the infection) were individually tested. The lines within the graphs represent the cut-off of each assay.

**Fig 5:**
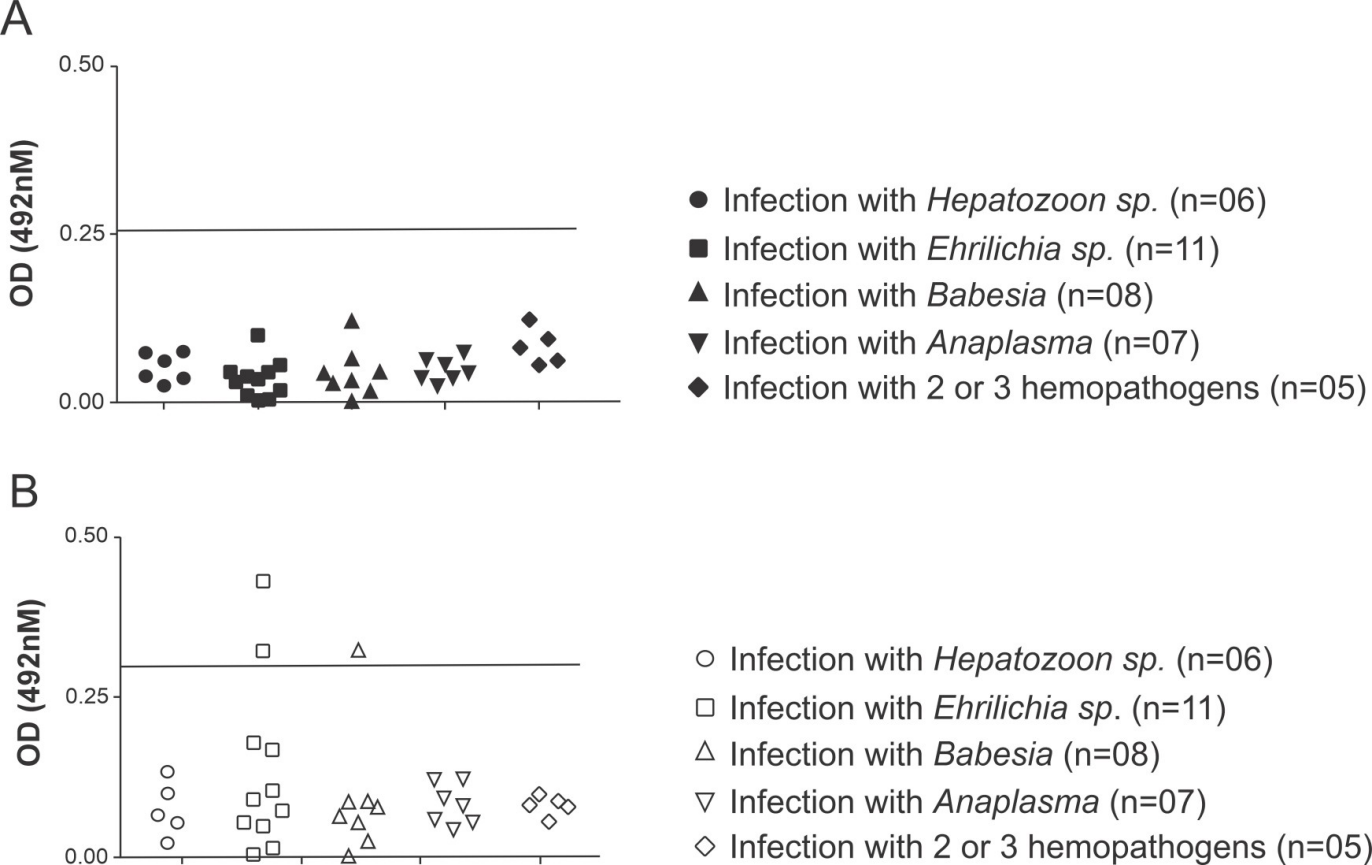
**Evaluation of the occurrence of cross-reactivity with the (A) LPG and (B) TLA-ELISAs with samples from dogs infected with other protozoa and bacteria of small animal medicine importance.** Serum samples from dogs infected with *Hepatozoon* sp., *Ehrlichia* sp., *Anaplasma* sp. and *Babesia* sp. were tested individually using the LPG-ELISA or TLA-ELISA.

When serum samples from dogs with confirmed infection by *T*. *cruzi* or *L*. *braziliensis* (with or without clinical signs of disease) were tested, no positive reactions could be seen by either LPG- or TLA-ELISA (**Fig 4**). However, when sera from dogs with confirmed infection by *Hepatozoon* sp., *Ehrlichia* sp., *Babesia* sp. or *Anaplasma* spp. were tested, the TLA-ELISA presented two positive cross reacting results (2/11–18.18%), one for a dog that was infected with *Ehrlichia* sp. and one for a *Babesia* sp.-infected dog (**Fig 5**).

### LPG-ELISA is able to detect subclinical *L*. *infantum* infection in dogs

**Fig 6:**
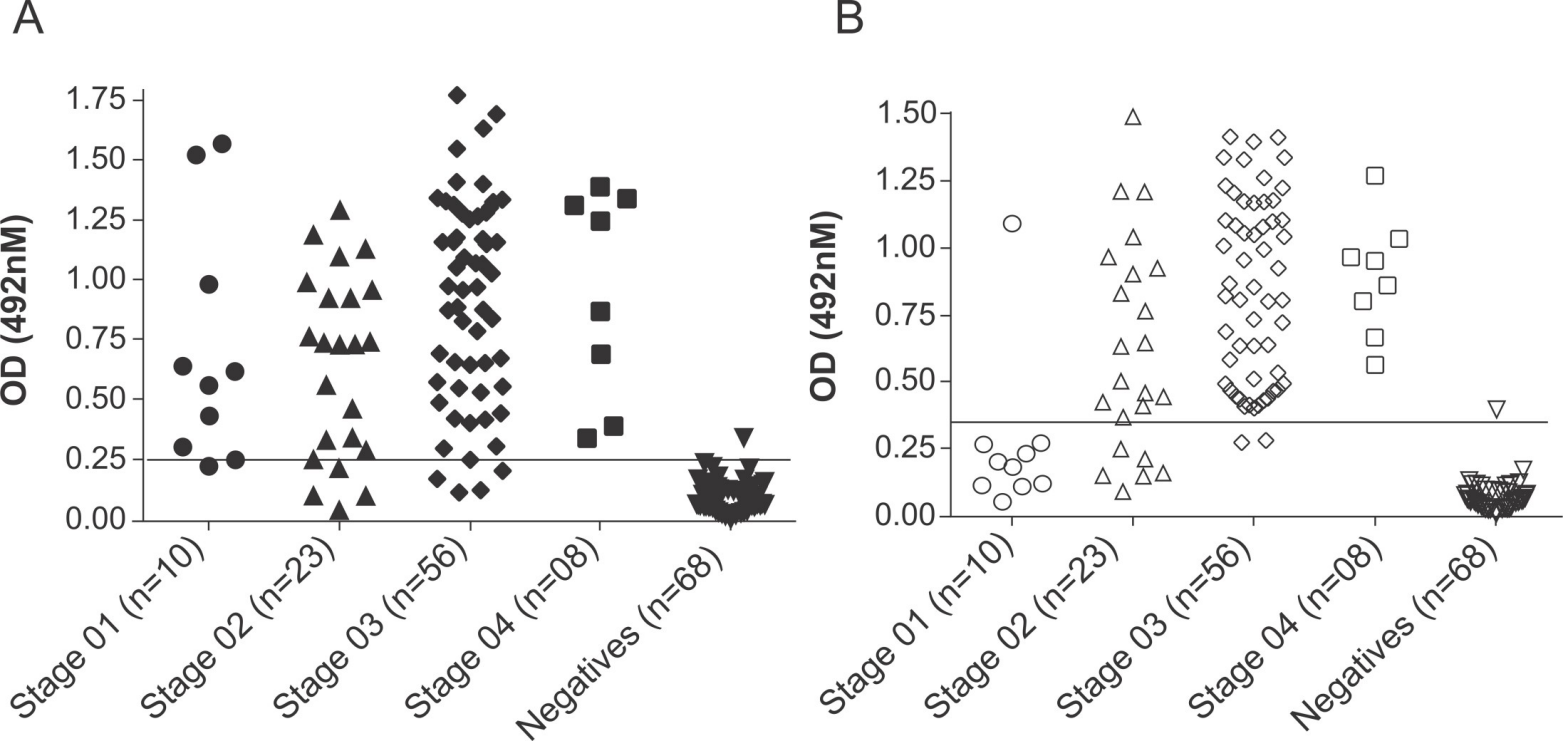
**Evaluation of the performance of (A) the LPG-ELISA and (B) the identification of *L*. *infantum*-specific immunoglobulins in dogs with different stages of canine visceral leishmaniasis.** The dogs underwent clinical staging based on their clinical signs and clinical pathology profiles. The lines within the graphs represent the established cut-off for each assay.

Regarding the ELISA tests carried out with serum samples from dogs with subclinical infection by *L*. *infantum*, the LPG-ELISA identified 9/10 (90%) as positive, while just one sample (1/9) was detected as positive by the TLA-ELISA (**Fig 6**). Both assays presented similar performances regarding animals in stages 2, 3 and 4 of the disease.

### The ELISA using LPG as antigen has a better diagnostic performance than the TR DPP -CVL immunochromatographic assay

The TR DPP-CVL immunochromatographic assay presented 10 false negative and 05 false positive results, while LPG-ELISA had 09 false negatives and 01 false positives. These results conferred a 93.1% specificity and 90.6% sensitivity for TR DPP, and LPG-ELISA consequently had higher validation parameters (98.5% specificity and 91.5% sensitivity). Regarding dogs with subclinical *L*. *infantum* infection, TR DPP-CVL was able to correctly diagnose as positive 06 dogs, and LPG-ELISA presented 09 positive results. Interestingly, the cross-reactions in samples from dogs experimentally infected with *T*. *cruzi* and in the acute phase of the infection were significant (60% positive results), and 03 of 13 animals that had a natural infection by *L*. *braziliensis* were positive when using TR DPP-CVL. None of the *T*. *cruzi* or *L*. *braziliensis* infected animals gave positive results for LPG-ELISA (**Table 3**).

**Table 3:** Comparison between LPG-ELISA and TR DPP-CVL immunochromatographic assay for the diagnosis of canine leishmaniasis. The same serum samples were tested in both assays. TR DPP-CVL procedures were conducted as recommended by the manufacturer.

Parameters	LPG-ELISA	TR DPP
	**POSITIVE AND NEGATIVE CONTROLS**	
False positives	1	5
False negatives	9	10
Specificity (%)	98.5	93.1
Sensitivity (%)	91.5	90.6
Positive Predictive Value (%)	98.9	94.5
Negative Predictive Value (%)	89.3	86.3
	**PERFORMANCE IN DIFFERENT CLINICAL STAGES**	
Stage 1 (subclinical infection)	90% positive (09/10)	60% positive (06/10)
	10% negative (01/10)	40% negative (04/10)
Stage 2	82.6% positive (19/23)	86.9% positive (20/23)
	17.3% negative (04/23)	13.1% negative (03/23)
Stage 3	92.8% positive (52/56)	94.6% positive (53/56)
	7.2% negative (04/56)	5.4% negative (03/56)
Stage 4	100% positive (8/8)	100% positive (8/8)
	**DETECTION OF CROSS-REACTIONS**	
*T*. *cruzi* experimental infection (acute phase)	0% positive (0/10)	60% positive (6/10)
	100% negative (10/10)	40% negative (4/10)
*T*. *cruzi* experimental infection (chronic phase)	0% positive (0/10)	0% positive (0/10)
	100% negative (10/10)	100% negative (10/10)
*L*. *braziliensis-*infected dogs	0% positive (0/13)	23% positive (3/13)
	100% negative (13/13)	77% positive (10/13)

## Discussion

There is still a demand for a commercially available immunoassay to diagnose CanL that is accurate enough to meet the requirements for good preventive veterinary medicine and public health actions. False-positive results can lead to the unnecessary treatment or even culling of non-infected dogs. On the other hand, tests that are not sensitive enough to detect infected subclinical dogs can lead to ineffective control of the disease, since these dogs would not be cared for and might act as reservoirs [32]. For this reason, in Brazil the official protocol recommended by the Ministry of Agriculture for the diagnosis of CanL is based on the application of two tests, a screening immunochromatographic assay with the recombinant rK28 protein as antigen (TR DPP-CVL), and a confirmatory ELISA with *L*. *major* total antigen (EIE). The need for two tests makes the control of the disease expensive and time-consuming. In this context, our proposal was to evaluate a non-protein LPG glycoconjugate as an antigen for the ELISA test, since these molecules are immunogenic, highly stable and broadly expressed by *L*. *infantum*.

The use of glycoconjugates as antigens for the development of vaccines or in immunodiagnostic assays has been reported [33–35]. *L*. *infantum* LPG has been previously characterized according to the number of sugars branching off the repeat unit motif; here, we chose type I LPG from Ba262 *L*. *infantum* strain, originally isolated from a dog. In the present study, our data show that LPG (and perhaps its PG motif) induced a significant humoral response, expressed by the intense recognition of this molecule by specific antibodies from infected dogs, while the negative controls did not. Supporting our reasoning, anti-*L*. *infantum* LPG antibodies have been previously found in human patients from areas endemic for leishmaniasis [33, 20]. Interestingly, these studies demonstrated that antibodies to *L*. *infantum* LPG were detected in patients without history of disease, which indicates subclinical infection.

The present LPG-ELISA exhibited 98.5% specificity and 91.5% sensitivity, while the TLA-ELISA, despite having the same specificity, achieved only 85.0% sensitivity. The accuracy levels of the LPG-ELISA and TLA-ELISA were 99.7% and 98.5%, respectively. A similar result was found by de Arruda et al. [38], who described a validation of two ELISAs produced by the governmental Brazilian Bio-Manguinhos, one of them using a *L*. *infantum* TLA as antigen, and the other one that became the official EIE for CanL control in the country. The authors described 91.85% specificity, 83.75% sensitivity and 91.7% accuracy for the *L*. *major* antigen (EIE) and 89.80% specificity, 82.69% sensitivity and 89.3% accuracy for the *L*. *infantum* [36]. Comparatively, the LPG-ELISA in our study was able to identify more infected dogs with fewer false-negative results since it presented higher sensitivity than that described for the EIE. Moreover, the literature reports similar specificity (92%) and sensitivity (92%) values for a LPG-ELISA in the diagnosis of human infection by *L*. *infantum* in African and Mediterranean countries [33, 20].

The LPG-ELISA reported herein also shows a relevant capacity of recognizing an antibody response to *L*. *infantum* in healthy dogs with subclinical infection. The difficult diagnosis of subclinically infected dogs has been one of the reasons for several studies on the immunodiagnostics of CanL, which have focused on the use of peptides and proteins developed in genomic, bioinformatics or proteomic studies to improve the sensitivity of serological tests for CanL [37]. In fact, Faria et al. [16], using multiepitope synthetic proteins, were able to identify subclinically infected dogs more effectively (80%) than the Brazilian official tests EIE (0%) and TR DPP-CVL (10%). Moreover, ELISAs with different recombinant proteins used as antigens presented positivities ranging from 23% to 65%, depending on the stage of the disease [16]. Mendes et al. [38] found 98% sensitivity and 99% specificity in an ELISA using a synthetic bi-epitope peptide, but their study did not mention the clinical staging of the dogs. One of the consequences of such variable specificities and sensitivities of different assays for CanL immunodiagnosis is that the seroprevalence in endemic areas can present marked fluctuations depending on the chosen assay [10]. In this study, we were able to include samples from 10 subclinically infected dogs that presented no alterations at the clinical examination and ancillary clinical pathology tests made by veterinarian infectologists.

The LPG used in the present study has been previously isolated and described as a highly stable molecule [36]. This high stability explains why the LPG-ELISA presented 97.5% repeatability and 99.7% reproducibility in the present study. These findings suggest that the LPG-ELISA is expected to present the same result for a given sample when tested several times by the same operator in one specific laboratory and likely even when tested by different laboratories and different operators. De Arruda et al. [36] pointed to the fact that an assay for CanL immunodiagnosis must have high reproducibility in order to be broadly used as a reference assay. A later study reported a positive predictive value (PPV) of 27.8% and a negative predictive value (NPV) of 99.1% when using a *L*. *infantum* TLA and 29.6% PPV and 99.3% NPV with a *L*. *major* TLA [39]. Our results show higher values for PPV and NPV, indicating that the LPG-ELISA can be used as a reliable tool for epidemiologic surveys. In addition, the procedure to purify *L*. *infantum* LPG has been shown to result in a high yield [21]. Such characteristics make the present *L*. *infantum* LPG a molecule that can be easily obtained and purified and that is capable of allowing a long validity span to commercial immunodiagnostic kits.

One of the procedures adopted by some countries for CanL control is the culling of *Leishmania*-infected dogs. However, in the human-dog interaction, dogs should not be considered irrelevant, since there is a strong link between owners and their companion animals, and their killing can cause intense emotional damage [40], and no scientific evidence supports that this procedure is effective in reducing the incidence of visceral leishmaniasis [41]. Taking into consideration that the LPG-ELISA presented a high capacity of diagnosis with minor cross reactivity, owing to its high specificity value, one can conclude that this assay will produce a very low frequency of false-positive results. Accordingly, considerable unnecessary culls of noninfected dogs can be avoided. The occurrence of cross-reactions with other pathogens that are endemic to the same areas as *Leishmania* is another problem that hinders the proper diagnosis of CanL [42]. The LPG-ELISA did not result in any cross-reactivity in the serum samples from dogs infected by *L*. *braziliensis*, *T*. *cruzi*, *Babesia* sp., *Ehrlichia* sp. or *Hepatozoon* sp. that were tested in this study, while the TLA-ELISA presented cross reactivity for *Ehrlichia* sp. (18.2% - 2/11) and *Babesia* sp. (12.5% - 1/8). Recently, Carvalho et al. [15] proposed an ELISA using a *L*. *infantum* LiHypA recombinant antigen, which conferred 100% sensitivity and 99% specificity for CanL diagnosis, but it exhibited cross reactivity with *B*. *canis*. A strategy used by various authors to avoid false-positive results is to standardize the serological test by highly diluting the serum samples, but such a procedure can reduce the sensitivity of the method. The standardization of our LPG- ELISA protocol included a broad range of serum dilutions and resulted in an assay that was free of cross reactions and false-positive results.

To improve specificity and reduce false-positive results due to cross-reactions, numerous studies have focused on the use of recombinant proteins as antigens in immunoassays for CanL. Two of those antigens, rK39 and rk26 *L*. *infantum*-derived recombinant proteins, were produced with the purpose of being purer antigens, thus containing a less diversity of epitopes that could promote reactions with antibodies produced in dogs infected with other pathogens [43, 44]. However, a study described that the use of rK39 and rK26 did not promote a significant increase in the specificity or sensitivity of serological assays when compared to *L*. *infantum* total antigen [45]. Comparing rK39 and rK26 with another recombinant antigen, rA2, in ELISA tests, Porrozzi et al. [46] found that, even though rA2 increased the test’s specificity, it still presented cross-reactivity with sera from *Leptospira interrogans*-infected dogs, while rK26 and rK39 presented cross-reactivity with sera from dogs infected with *L*. *braziliensis*. Conversely, the present LPG-ELISA showed high specificity and sensitivity, and no cross-reactions were observed when testing serum samples from dogs infected with other infectious agents, even those with *L*. *braziliensis*. The absence of cross reactivity by the sera from dogs infected with *L*. *braziliensis* in the LPG-ELISA could be because *L*. *braziliensis* expresses 10 to 20-fold less LPG than the other *Leishmania* species [21]. In this sense, the immune system of *L*. *braziliensis-*infected dogs would be less exposed to LPG interactions; consequently, there would be less immunogenic activation and weaker antibody responses to this specific molecule. Nevertheless, it should be mentioned that *L*. *braziliensis* LPG is very similar to *L*. *infantum* type 1 LPG [27].

One of the most notable results of the present work is that *L*. *infantum*-infected dogs presenting no clinical signs of the disease, herein classified as having subclinical infection, were expressively identified as positive by the LPG-ELISA (90% - 9/10). In contrast, the TLA-ELISA diagnosed only one of those ten dogs as positive (10%). The proper diagnosis of subclinically infected dogs is very important for the control of leishmaniasis in endemic areas, since these dogs can remain untreated or unfollowed. Susceptible subclinically infected dogs can develop active infection, representing a source of infection for other dogs and humans. Such dogs, even with a recent infection, produce specific antibodies in high levels, before developing clinical signs and would also be characterized as having subclinical infection [47]. *Leishmania*-specific humoral responses in susceptible dogs are characterized by high levels of specific immunoglobulins [48]. However, such abundant immunoglobulins are non-protective and are instead associated with active infection, intense disease development and presentation of clinical signs in dogs [47, 48]. On the other hand, resistant dogs remain clinically healthy and have a small or frequently undetectable parasite load; however, they can present fluctuating and low levels of anti-*Leishmania* immunoglobulins [46]. Indeed, the immunodiagnosis of dogs with unapparent infection is a difficult task and several assays have failed to be sensitive enough to detect infection in such dogs. By using rK39 as an antigen in ELISA tests, De Lima et al. [49] found that the assay was 100% specific for *L*. *infantum* diagnosis in clinically diseased dogs, but it failed to detect nine dogs with subclinical infection. Similarly, De Carvalho et al. [50] showed that 18.7% of dogs that were PCR-positive for *L*. *infantum* DNA were diagnosed negative in the official two-test Brazilian protocol. Another study showed that the rLiHypA antigen provided 100% detection of subclinically infected dogs, but the test’s accuracy was impaired by its cross-reaction with babesiosis [15].

In a recent study, Figueiredo et al. [51] evaluated the performance of the previously validated TR DPP-CVL in samples taken from 1446 dogs, having found a sensitivity of 89% and a specificity of 70%, and observed positive results in only 75% of the subclinically infected (“asymptomatic”) dogs. We found a similar result in the present study, where only six out of ten (60%) dogs with subclinical *L*. *infantum* infection tested positive in the TR DPP-CVL, while the LPG-ELISA was able to detect nine of those dogs (90%). Even though we have compared two assays using different platforms, our results can suggest that a similar immunochromatographic assay using *L*. *infantum* LPG as antigen might as well result in a very sensitive and specific diagnostic test. In addition, an interesting finding was that 60% of dogs that were in the acute phase of an experimental infection with *T*. *cruzi* presented positive results in the TR DPP-CVL, but the same dogs became negative when in the chronic phase of the trypanosomiasis. A possible explanation for this result is that the TR DPP-CVL uses the Staphylococcal protein A conjugated to colloidal gold as a development reagent. It has been described that protein A has affinity not only for IgG but also for IgM [52]; in this way, large amounts of IgM produced after the experimental infection with *T*. *cruzi* might have been a source of cross-reaction in the TR DPP-CVL. It is noteworthy that low affinity IgM produced by the B1 subpopulation of B-lymphocytes can be related to cross reactions [53].

When serum samples taken from endemic area’s negative dogs were used to determine a regional cut-off, the sensitivity of LPG-ELISA dropped to 84.6%, since the employment of a higher cut-off led to the occurrence of 15 false-negative results among a total of 97 samples. These results express an example from a local situation and represent a methodology used by many clinical laboratories to establish regional cut-offs. However such an approach should not be applied to the overall evaluation of the present LPG-ELISA´s parameters standardization. Indeed, samples from an endemic area cannot be trusted as truly negative due to the fact that infected dogs are not always positive in parasitological or molecular assays, since the parasite may be present in other tissues than the ones that were sampled [23]. In this way, the use of negative samples from non-endemic areas represented the overall capacity of the present LPG-ELISA to discriminate between truly infected or non-infected dogs.

In conclusion, *L*. *infantum*-derived LPG presented high efficacy in the detection of specific immunoglobulins in dogs infected with the parasite when used in an ELISA-based diagnostic test. The LPG-ELISA showed no cross-reactions with sera from dogs infected with other pathogens and was able to identify 90% of the samples from dogs with subclinical infection. Because of the high stability of the LPG molecule and its high yield and simple and cheap purification methodology, it is a promising molecule for a highly accurate immunodiagnosis of CanL.

## Supporting information

S1 Table Individual optical density results of the negative and positive controls used for the standardization of the LPG-ELISA. (PDF)

S1 Fig LPG-ELISA results for serum samples taken from dogs from an endemic area and presenting negative results in molecular and parasitological assays. (A) Distribution of LPG-ELISA OD results for the 30 endemic area negative samples at the LPG-ELISA. (B) Distribution of the optical density results of the positive and negative controls used for the standardization of the ELISA, now considering the cut-off established with the endemic area negative samples. The bar indicates the cut-off calculated with these 30 negative samples from the endemic area.
(PDF)
