# Supplementary material for: Leishmania infantum-derived lipophosphoglycan as an antigen in the accurate serodiagnosis of canine leishmaniasis
Source: PLoS Negl Trop Dis. 2019 Sep 12;13(9):e0007720. doi: 10.1371/journal.pntd.0007720 (PMC6759188; doi:10.1371/journal.pntd.0007720)
Supplement: S1 Table — (PDF) [file pntd.0007720.s001.pdf]

S1 Table – Individual optical density results of the negative and positive controls used for the standardization of the LPG-ELISA.

LPG-ELISA OD (492 nM) of Positive controls (n=97)					
SAMPLE ID	OD	SAMPLE ID	OD	SAMPLE ID	OD
1	0.049	34	0.621	67	1.094
2	0.102	35	0.643	68	1.127
3	0.105	36	0.649	69	1.151
4	0.120	37	0.654	70	1.155
5	0.129	38	0.657	71	1.157
6	0.175	39	0.674	72	1.159
7	0.209	40	0.694	73	1.169
8	0.219	41	0.696	74	1.175
9	0.229	42	0.732	75	1.187
10	0.253	43	0.733	76	1.244
11	0.254	44	0.738	77	1.252
12	0.254	45	0.743	78	1.265
13	0.300	46	0.763	79	1.278
14	0.300	47	0.787	80	1.282
15	0.308	48	0.827	81	1.291
16	0.312	49	0.840	82	1.309
17	0.336	50	0.870	83	1.309
18	0.342	51	0.874	84	1.322
19	0.345	52	0.878	85	1.325
20	0.393	53	0.886	86	1.333
21	0.406	54	0.920	87	1.338
22	0.421	55	0.922	88	1.338
23	0.424	56	0.956	89	1.387
24	0.436	57	0.957	90	1.397
25	0.446	58	0.968	91	1.407
26	0.465	59	0.975	92	1.519
27	0.491	60	0.983	93	1.543
28	0.533	61	0.989	94	1.565
29	0.551	62	1.027	95	1.629
30	0.559	63	1.052	96	1.686
31	0.561	64	1.064	97	1.765
32	0.564	65	1.070		
33	0.576	66	1.094		

LPG-ELISA OD (492 nM) of Negative controls (n=68)

SAMPLE ID	OD	SAMPLE ID	OD
98	0.009	132	0.072
99	0.013	133	0.073
100	0.017	134	0.077
101	0.023	135	0.080
102	0.024	136	0.086
103	0.024	137	0.087
104	0.026	138	0.091
105	0.030	139	0.095
106	0.032	140	0.098
107	0.034	141	0.102
108	0.041	142	0.107
109	0.042	143	0.107
110	0.042	144	0.111
111	0.043	145	0.114
112	0.043	146	0.119
113	0.046	147	0.124
114	0.053	148	0.129
115	0.058	149	0.131
116	0.058	150	0.134
117	0.059	151	0.140
118	0.060	152	0.140
119	0.061	153	0.143
120	0.061	154	0.145
121	0.062	155	0.148
122	0.064	156	0.152
123	0.066	157	0.161
124	0.067	158	0.163
125	0.067	159	0.170
126	0.069	160	0.174
127	0.069	161	0.188
128	0.069	162	0.214
129	0.070	163	0.224
130	0.071	164	0.240
131	0.072	165	0.344
