# Supplementary material for: Leishmania infantum-derived lipophosphoglycan as an antigen in the accurate serodiagnosis of canine leishmaniasis
Source: PLoS Negl Trop Dis. 2019 Sep 12;13(9):e0007720. doi: 10.1371/journal.pntd.0007720 (PMC6759188; doi:10.1371/journal.pntd.0007720)

S1 Figure – LPG-ELISA results for serum samples taken from dogs from an endemic area and presenting negative results in molecular and parasitological assays. (A) Distribution of LPG-ELISA OD results for the 30 endemic area negative samples at the LPG-ELISA. **(B)** Distribution of the optical density results of the positive and negative controls used for the standardization of the ELISA, now considering the cut-off established with the endemic area negative samples. The bar indicates the cut-off calculated with these 30 negative samples from the endemic area.

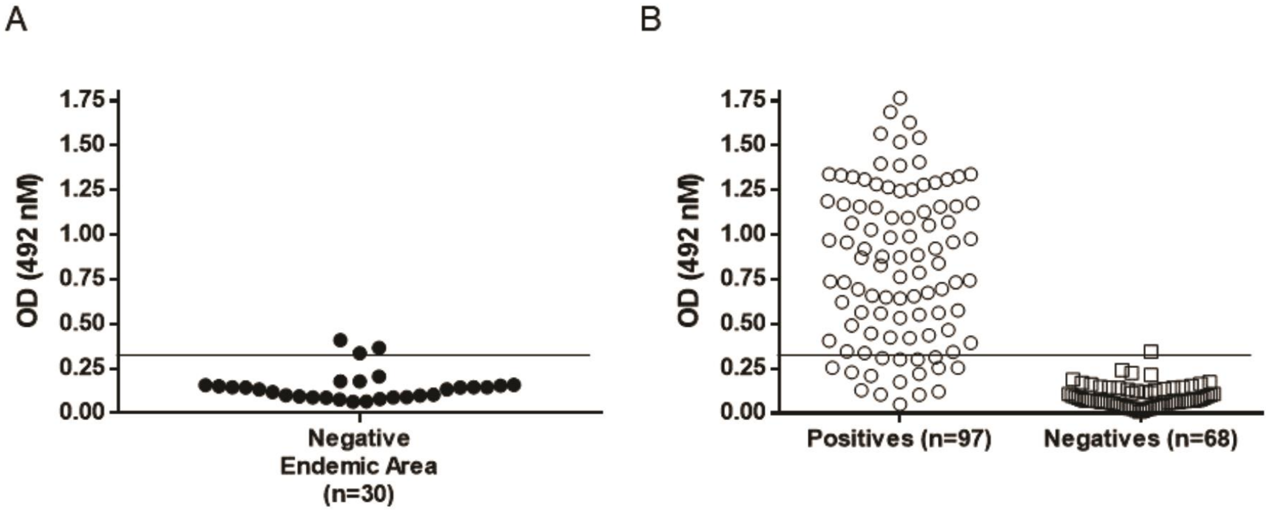

Supplement: S1 Fig — (A) Distribution of LPG-ELISA OD results for the 30 endemic area negative samples at the LPG-ELISA. (B) Distribution of the optical density results of the positive and negative controls used for the standardization of the ELISA, now considering the cut-off established with the endemic area negative samples. The bar indicates the cut-off calculated with these 30 negative samples from the endemic area. (PDF) [file pntd.0007720.s002.pdf]
